# P-2129. Prolonged infusion of β-lactams for Enterobacterales bacteremia in high-risk neutropenic patients: Does it improve outcomes?

**DOI:** 10.1093/ofid/ofaf695.2293

**Published:** 2026-01-11

**Authors:** Fabián Herrera, Diego Torres, Lucas Tula, Noelia Mañez, María Laura Pereyra, Lorena Berruezo, José Benso, Ana Laborde, Nadia Suchowiercha, Andrea Nenna, Jorge López Camelo, Rocío Gago, Nadia Ailen Fernández, Verónica Fernández, María Luz González Ibañez, Inés Roccia Rossi, Vanesa Soto, Natalín Grippo, Miriam Blanco, Mariángeles Visús, Natalia Azula, Ruth Carbone, Magdalena Pennini, Mariana Reynaldi, María Laura Chaves, Fernando Pasteran, Alejandra Corso, Melina Rapoport, Alberto Carena

**Affiliations:** Centro de Educación Médica e Investigaciones Clínicas, CEMIC, BUENOS AIRES, Ciudad Autonoma de Buenos Aires, Argentina; Centro de Educación Médica e Investigaciones Clínicas, CEMIC, BUENOS AIRES, Ciudad Autonoma de Buenos Aires, Argentina; Hospital El Cruce, Florencio Varela, Buenos Aires, Argentina; Hospital Italiano de Buenos Aires, Sede Central., Buenos Aires, Ciudad Autonoma de Buenos Aires, Argentina; Hospital Universitario Austral, Pilar, Buenos Aires, Argentina; HIGA "Dr. Rodolfo Rossi", La Plata, Buenos Aires, Argentina; Hospital Italiano de San Justo, San Justp, Buenos Aires, Argentina; FUNDALEU, Buenos Aires, Ciudad Autonoma de Buenos Aires, Argentina; HIGA "Gral. San Martín", La Plata, Buenos Aires, Argentina; Hospital Municipal de Oncología Marie Curie, Buenos Aires, Ciudad Autonoma de Buenos Aires, Argentina; CEMIC, Buenos Aires, Ciudad Autonoma de Buenos Aires, Argentina; Hospital Universitario Austral, Pilar, Buenos Aires, Argentina; HIGA Rodolfo Rossi, La Plata, Buenos Aires, Argentina; Hospital Italiano de San Justo, San Justp, Buenos Aires, Argentina; FUNDALEU, Buenos Aires, Ciudad Autonoma de Buenos Aires, Argentina; HIGA "Gral. San Martín", La Plata, Buenos Aires, Argentina; Hospital Municipal de Oncología "Marie Curie", Buenos Aires, Buenos Aires, Argentina; CEMIC, Buenos Aires, Ciudad Autonoma de Buenos Aires, Argentina; Hospital El Cruce, Florencio Varela, Buenos Aires, Argentina; Hospital Italiano de Buenos Aires, Sede Central., Buenos Aires, Ciudad Autonoma de Buenos Aires, Argentina; Hospital Universitario Austral, Pilar, Buenos Aires, Argentina; HIGA "Dr. Rodolfo Rossi", La Plata, Buenos Aires, Argentina; CEI Dr Stamboulian, Buenos Aires, Ciudad Autonoma de Buenos Aires, Argentina; HIGA Gral. San Martín, La Plata, Buenos Aires, Argentina; Hospital Municipal de Oncología Marie Curie, Buenos Aires, Ciudad Autonoma de Buenos Aires, Argentina; ANLIS-Malbrán, Buenos Aires, Ciudad Autonoma de Buenos Aires, Argentina; ANLIS-Malbrán, Buenos Aires, Ciudad Autonoma de Buenos Aires, Argentina; ANLIS-Malbrán, Buenos Aires, Ciudad Autonoma de Buenos Aires, Argentina; Hospital Universitario CEMIC, Buenos Aires, Ciudad Autonoma de Buenos Aires, Argentina

## Abstract

**Background:**

No evidences have been reported on the clinical benefits of prolonged infusion (PI) of β-lactams in febrile neutropenic patients (FNP) with Enterobacterales bacteremia (EB).

**Methods:**

A prospective observational multicenter study was conducted in 9 referral academic centers in Argentina between February 2019 and December 2024. The first episodes of EB in high-risk FNP who received appropriate empirical treatment (AET) with piperacillin-tazobactam (PT), cefepime (C), or meropenem (M) were included. Clinical, epidemiological, and outcome variables were compared in patients receiving either standard infusion (SI) or PI AET. A propensity score (PS) to balance baseline covariates was used. Adjusted conditional multivariate logistic regression analysis to PS was used to identify independent risk factors for 30-day mortality.

**Results:**

201 patients were included (116 SI and 85 PI). Median age was 49 years (IQR: 35-63), and the most common underlying diseases were acute leukemia (61.69%) and lymphoma (18.91%). The microorganisms most frequently observed in both groups were *E. coli* (58.71%) and *Klebsiella* spp. (32.84%). Bacteremia presented with a clinical source in 58.82% of PI cases vs. 55.17% of SI cases, p = 0.60. Hypotension and shock were present in PI vs. SI in 28.4% vs. 25%, p=0.60, and 17.65% vs. 14.66%, p=0.56, respectively. Intensive care unit admission and multiorgan failure occurred in PI vs. SI: 18.82% vs. 12.07%, p = 0.56, and 16.47% vs. 11.21%, p = 0.28, respectively. Seven-day

clinical response between IP and IB was 90.59% vs. 93.10%, p = 0.51. Thirty-day overall mortality and EB-related mortality were 14.12% vs. 10.34% (p = 0.41) and 8.24% vs. 3.45% (p = 0.20) for PI and SI, respectively. Septic shock was the risk factor associated with 30-day mortality (OR: 11.65, 95% CI, 3.66-36.91, p < 0.001), while 7-day clinical response was a protective factor for survival (OR: 0.06, 95% CI, 0.01-0.24, p < 0.001). PI did not correlate with impact on mortality (OR: 1.78, 95% CI: 0.54-5.79, p = 0.34).

**Conclusion:**

In our high-risk FNP cohort with EB, AET with PI did not improve outcomes. However, randomized trials are needed to confirm the study findings.

**Disclosures:**

Alberto Carena, n/a, Roche Diagnostics: Medical affairs lead

